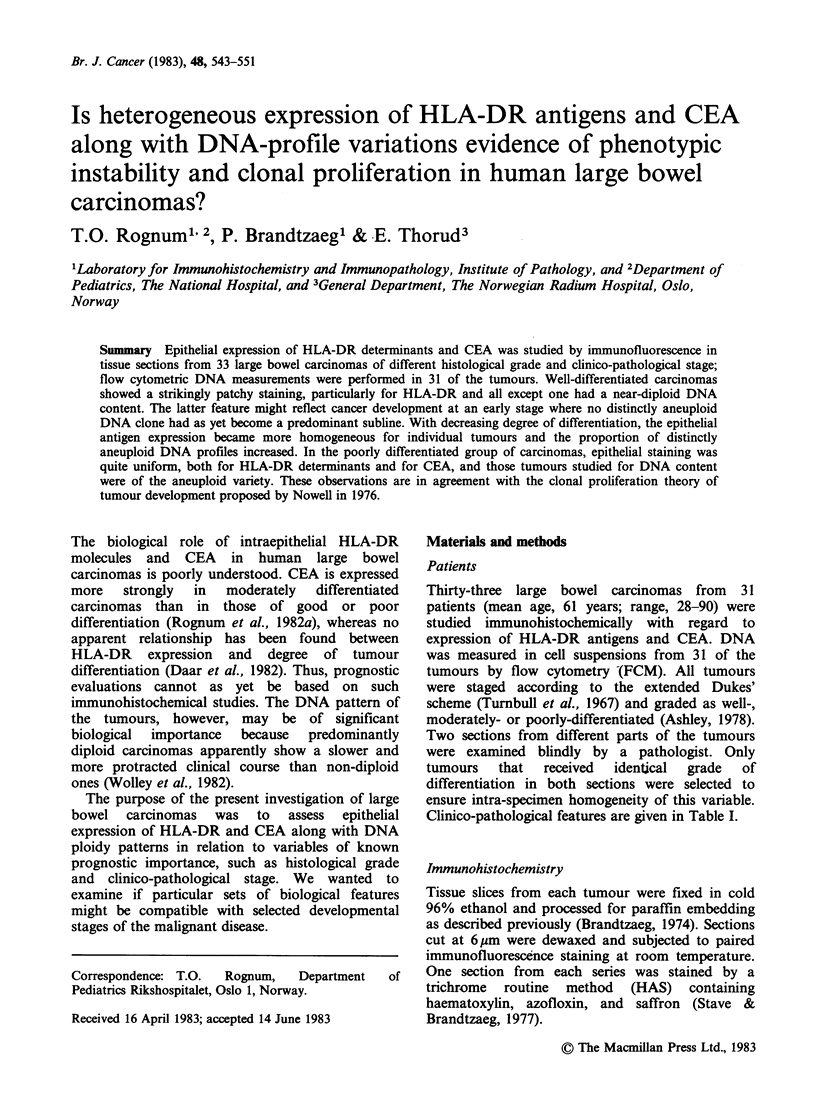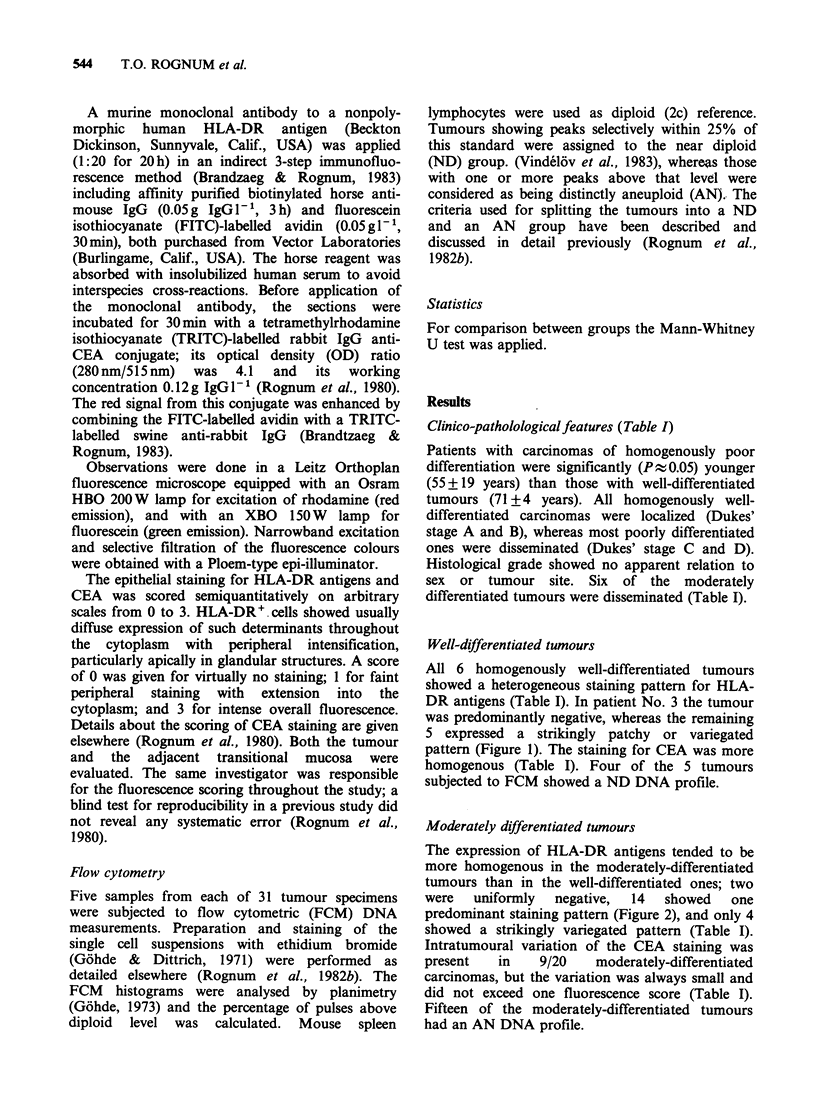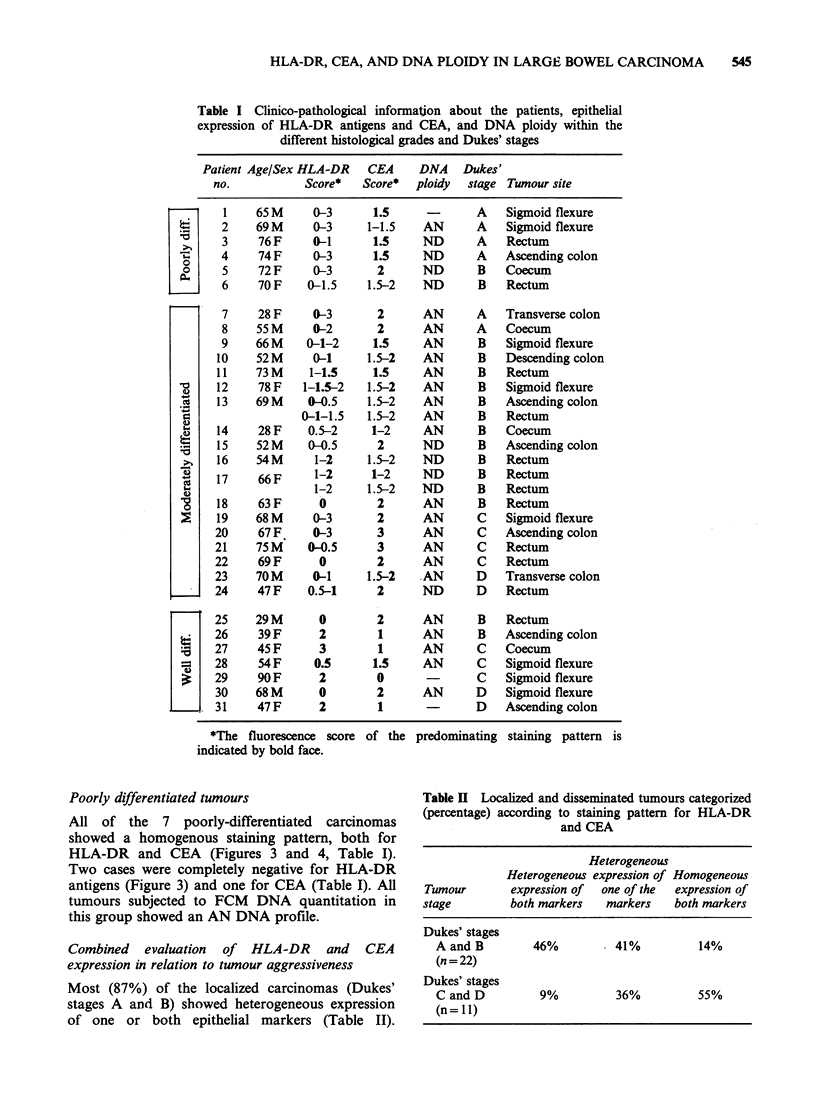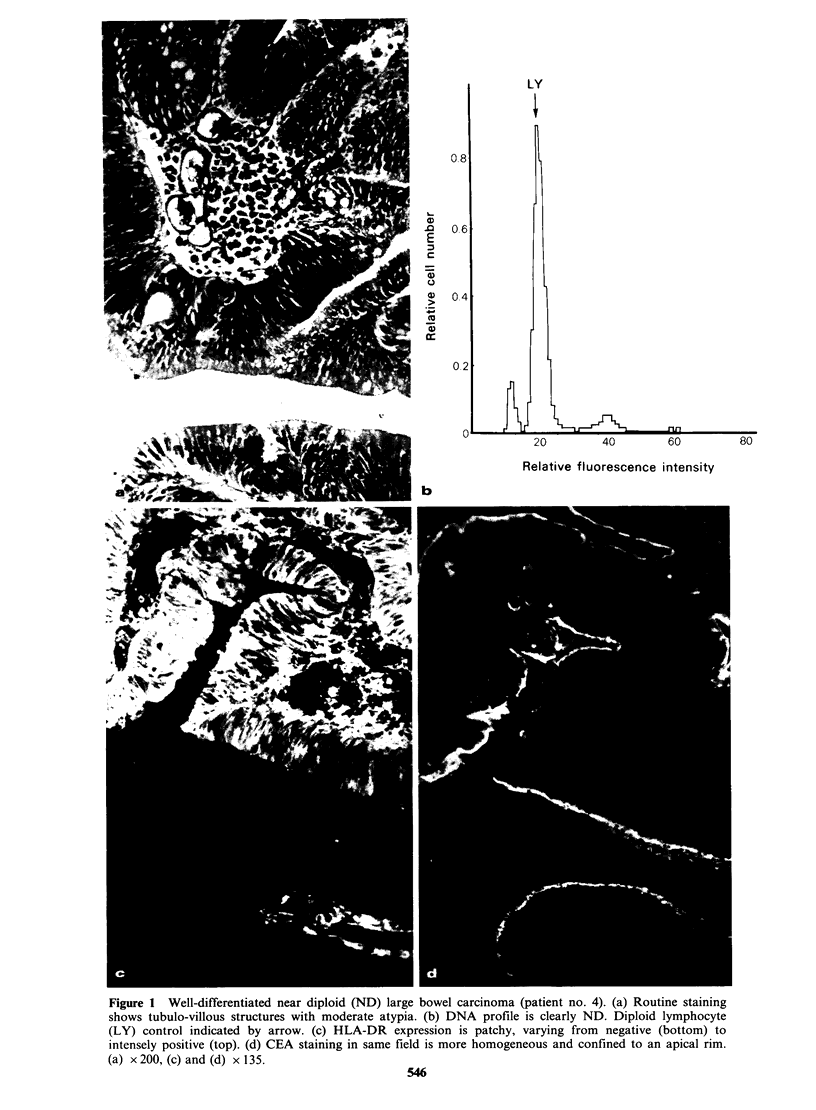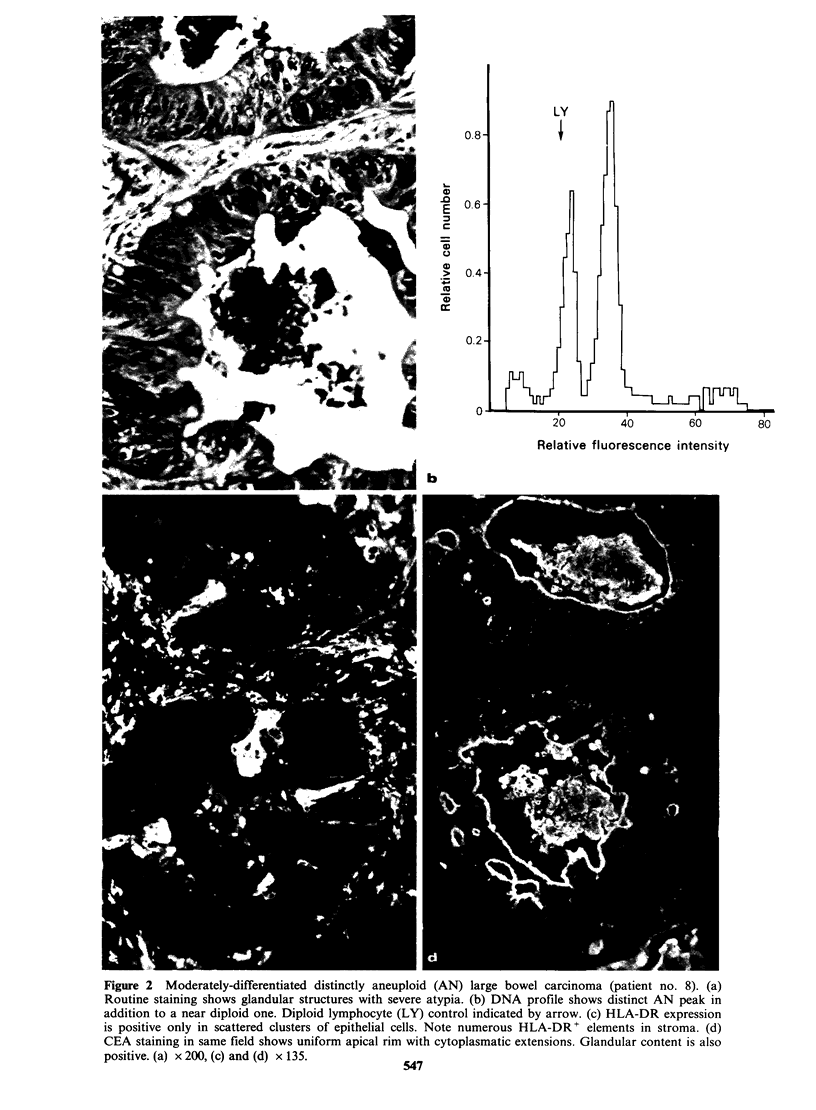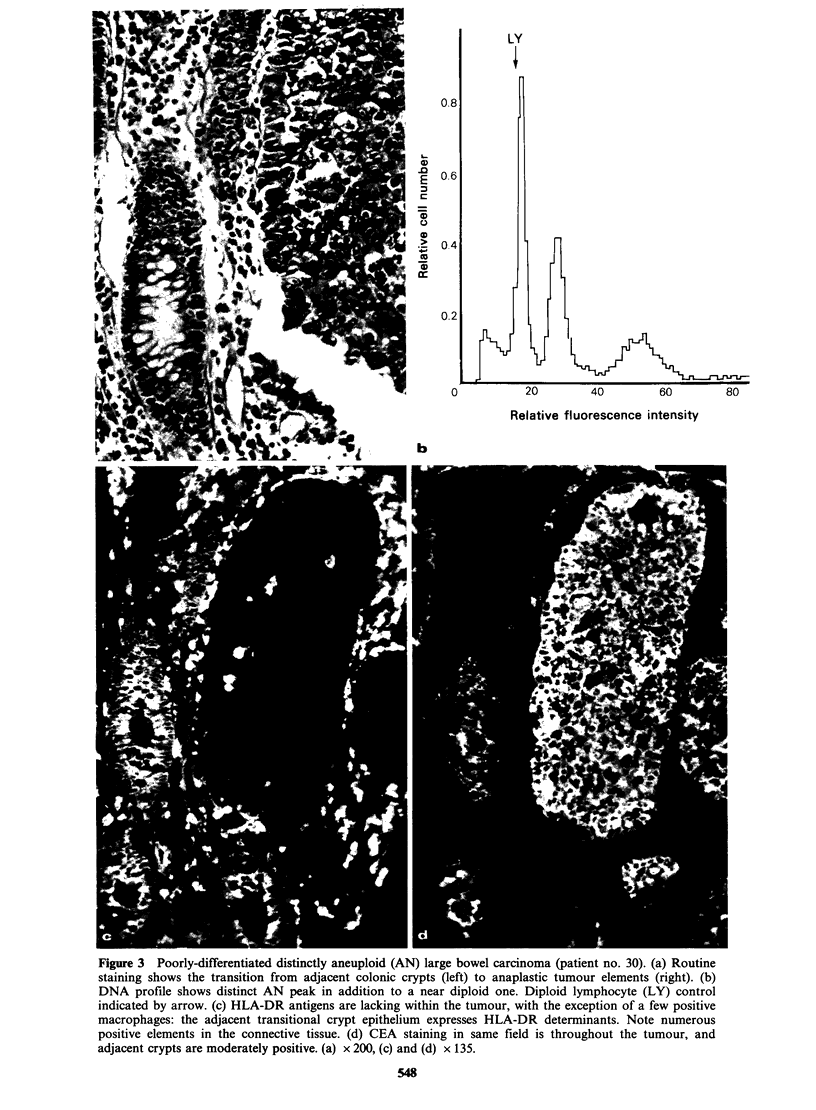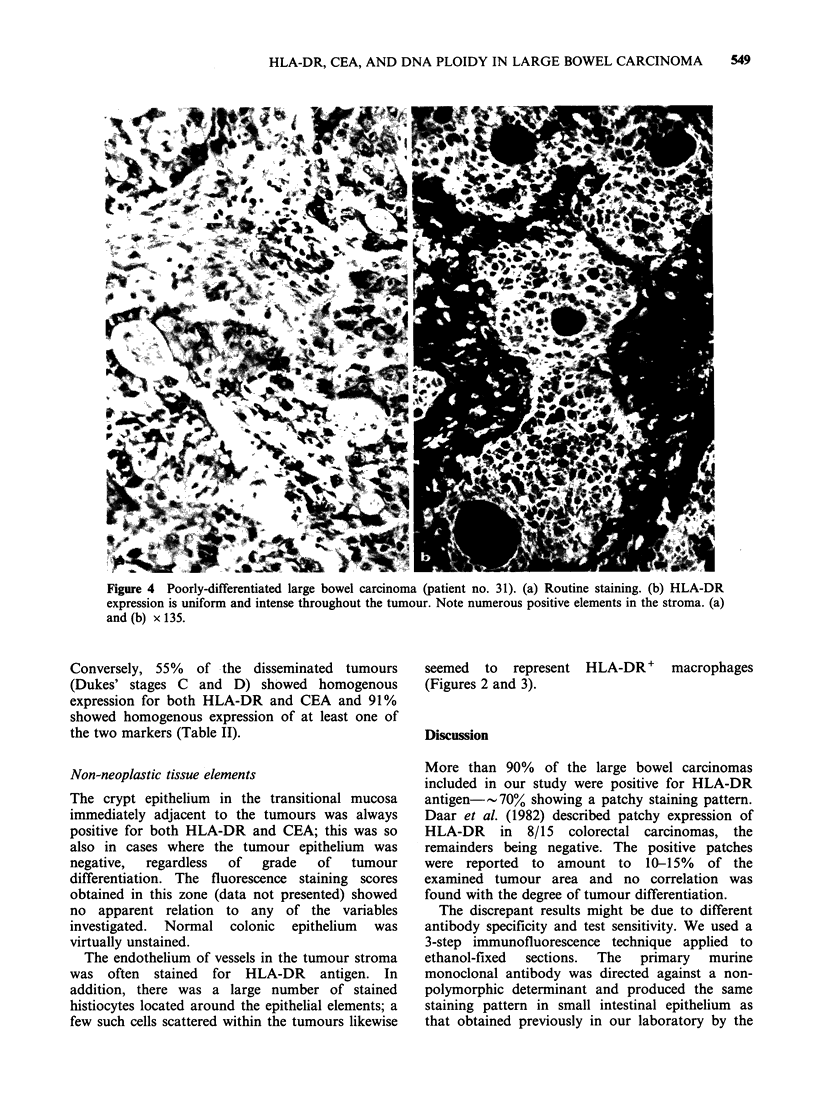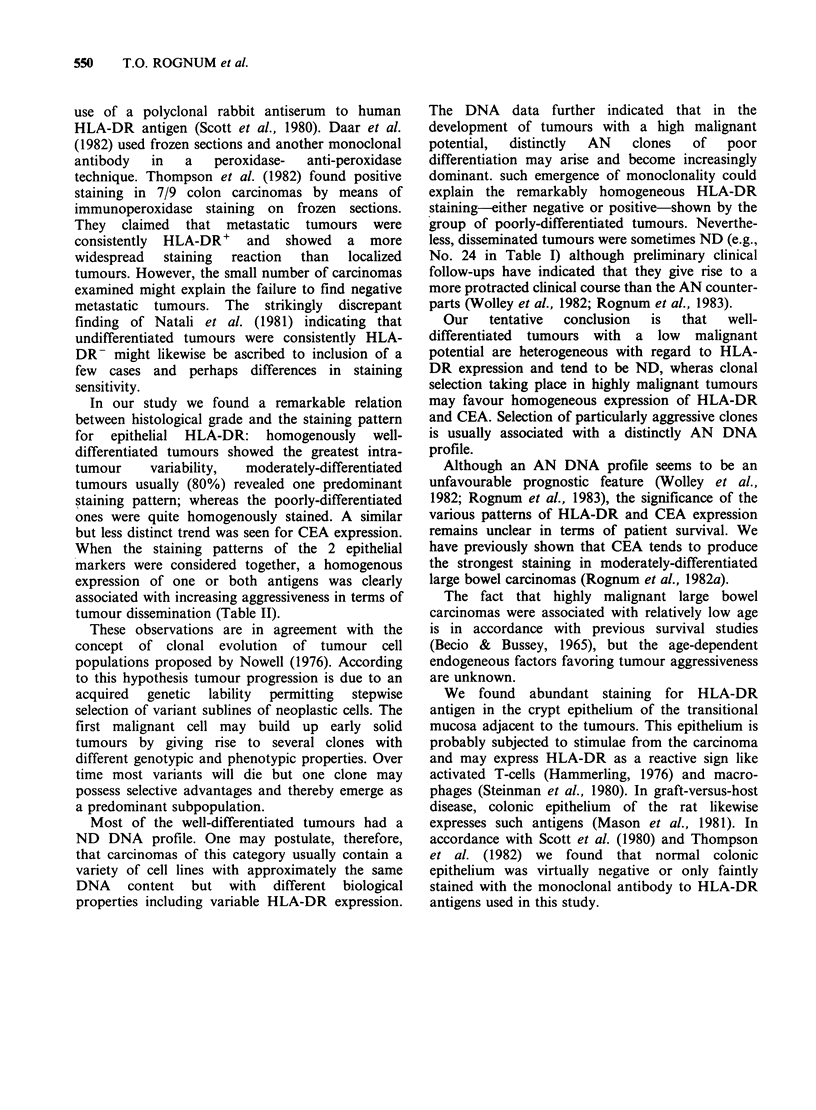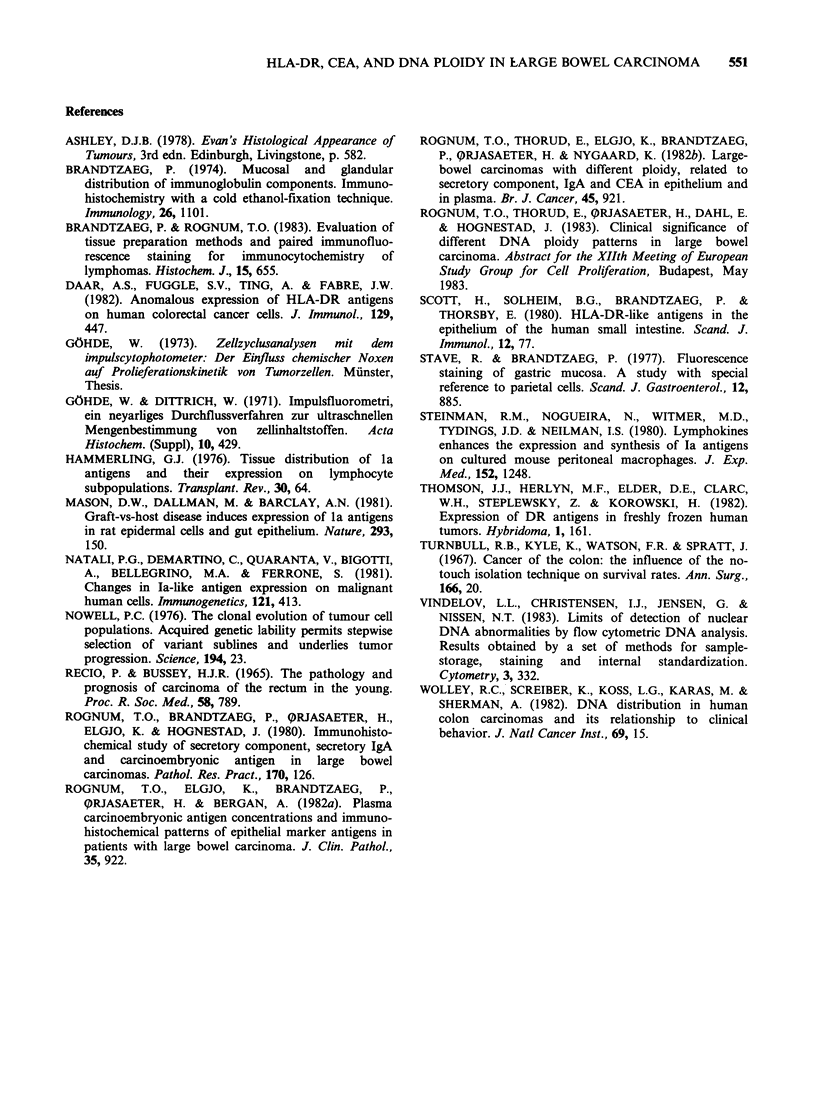# Is heterogeneous expression of HLA-dr antigens and CEA along with DNA-profile variations evidence of phenotypic instability and clonal proliferation in human large bowel carcinomas?

**DOI:** 10.1038/bjc.1983.227

**Published:** 1983-10

**Authors:** T. O. Rognum, P. Brandtzaeg, E. Thorud

## Abstract

**Images:**


*Br. J. Cancer* (1983), **48**, 543–551

### Is heterogeneous expression of HLA-DR antigens and CEA along with DNA-profile variations evidence of phenotypic instability and clonal proliferation in human large bowel carcinomas?

T.O. Rognum[1, 2], P. Brandtzaeg[1] & E. Thorud[3]

[1]*Laboratory for Immunohistochemistry and Immunopathology, Institute of Pathology, and* [2]*Department of Pediatrics, The National Hospital, and* [3]*General Department, The Norwegian Radium Hospital, Oslo, Norway*

**Summary** Epithelial expression of HLA-DR determinants and CEA was studied by immunofluorescence in tissue sections from 33 large bowel carcinomas of different histological grade and clinico-pathological stage; flow cytometric DNA measurements were performed in 31 of the tumours. Well-differentiated carcinomas showed a strikingly patchy staining, particularly for HLA-DR and all except one had a near-diploid DNA content. The latter feature might reflect cancer development at an early stage where no distinctly aneuploid DNA clone had as yet become a predominant subline. With decreasing degree of differentiation, the epithelial antigen expression became more homogeneous for individual tumours and the proportion of distinctly aneuploid DNA profiles increased. In the poorly differentiated group of carcinomas, epithelial staining was quite uniform, both for HLA-DR determinants and for CEA, and those tumours studied for DNA content were of the aneuploid variety. These observations are in agreement with the clonal proliferation theory of tumour development proposed by Nowell in 1976.

The biological role of intraepithelial HLA-DR molecules and CEA in human large bowel carcinomas is poorly understood. CEA is expressed more strongly in moderately differentiated carcinomas than in those of good or poor differentiation (Rognum *et al.*, 1982*a*), whereas no apparent relationship has been found between HLA-DR expression and degree of tumour differentiation (Daar *et al.*, 1982). Thus, prognostic evaluations cannot as yet be based on such immunohistochemical studies. The DNA pattern of the tumours, however, may be of significant biological importance because predominantly diploid carcinomas apparently show a slower and more protracted clinical course than non-diploid ones (Wolley *et al.*, 1982).

The purpose of the present investigation of large bowel carcinomas was to assess epithelial expression of HLA-DR and CEA along with DNA ploidy patterns in relation to variables of known prognostic importance, such as histological grade and clinico-pathological stage. We wanted to examine if particular sets of biological features might be compatible with selected developmental stages of the malignant disease.

Correspondence: T.O. Rognum, Department of Pediatrics Rikshospitalet, Oslo 1, Norway.

Received 16 April 1983; accepted 14 June 1983

### Materials and methods

### Patients

Thirty-three large bowel carcinomas from 31 patients (mean age, 61 years; range, 28–90) were studied immunohistochemically with regard to expression of HLA-DR antigens and CEA. DNA was measured in cell suspensions from 31 of the tumours by flow cytometry (FCM). All tumours were staged according to the extended Dukes' scheme (Turnbull *et al.*, 1967) and graded as well-, moderately- or poorly-differentiated (Ashley, 1978). Two sections from different parts of the tumours were examined blindly by a pathologist. Only tumours that received identical grade of differentiation in both sections were selected to ensure intra-specimen homogeneity of this variable. Clinico-pathological features are given in Table I.

### Immunohistochemistry

Tissue slices from each tumour were fixed in cold 96% ethanol and processed for paraffin embedding as described previously (Brandtzaeg, 1974). Sections cut at 6 $\mu$m were dewaxed and subjected to paired immunofluorescence staining at room temperature. One section from each series was stained by a trichrome routine method (HAS) containing haematoxylin, azofloxin, and saffron (Stave & Brandtzaeg, 1977).

© The Macmillan Press Ltd., 1983

A murine monoclonal antibody to a nonpolymorphic human HLA-DR antigen (Beckton Dickinson, Sunnyvale, Calif., USA) was applied (1:20 for 20 h) in an indirect 3-step immunofluorescence method (Brandzaeg & Rognum, 1983) including affinity purified biotinylated horse anti-mouse IgG (0.05 g $IgG l^{-1}$, 3 h) and fluorescein isothiocyanate (FITC)-labelled avidin (0.05 $g l^{-1}$, 30 min), both purchased from Vector Laboratories (Burlingame, Calif., USA). The horse reagent was absorbed with insolubilized human serum to avoid interspecies cross-reactions. Before application of the monoclonal antibody, the sections were incubated for 30 min with a tetramethylrhodamine isothiocyanate (TRITC)-labelled rabbit IgG anti-CEA conjugate; its optical density (OD) ratio (280 nm/515 nm) was 4.1 and its working concentration 0.12 g $IgG l^{-1}$ (Rognum *et al.*, 1980). The red signal from this conjugate was enhanced by combining the FITC-labelled avidin with a TRITC-labelled swine anti-rabbit IgG (Brandtzaeg & Rognum, 1983).

Observations were done in a Leitz Orthoplan fluorescence microscope equipped with an Osram HBO 200 W lamp for excitation of rhodamine (red emission), and with an XBO 150 W lamp for fluorescein (green emission). Narrowband excitation and selective filtration of the fluorescence colours were obtained with a Ploem-type epi-illuminator.

The epithelial staining for HLA-DR antigens and CEA was scored semiquantitatively on arbitrary scales from 0 to 3. $HLA-DR^{+}$ cells showed usually diffuse expression of such determinants throughout the cytoplasm with peripheral intensification, particularly apically in glandular structures. A score of 0 was given for virtually no staining; 1 for faint peripheral staining with extension into the cytoplasm; and 3 for intense overall fluorescence. Details about the scoring of CEA staining are given elsewhere (Rognum *et al.*, 1980). Both the tumour and the adjacent transitional mucosa were evaluated. The same investigator was responsible for the fluorescence scoring throughout the study; a blind test for reproducibility in a previous study did not reveal any systematic error (Rognum *et al.*, 1980).

### *Flow cytometry*

Five samples from each of 31 tumour specimens were subjected to flow cytometric (FCM) DNA measurements. Preparation and staining of the single cell suspensions with ethidium bromide (Göhde & Dittrich, 1971) were performed as detailed elsewhere (Rognum *et al.*, 1982*b*). The FCM histograms were analysed by planimetry (Göhde, 1973) and the percentage of pulses above diploid level was calculated. Mouse spleen lymphocytes were used as diploid (2c) reference. Tumours showing peaks selectively within 25% of this standard were assigned to the near diploid (ND) group. (Vindélöv *et al.*, 1983), whereas those with one or more peaks above that level were considered as being distinctly aneuploid (AN). The criteria used for splitting the tumours into a ND and an AN group have been described and discussed in detail previously (Rognum *et al.*, 1982*b*).

### *Statistics*

For comparison between groups the Mann-Whitney U test was applied.

### Results

### *Clinico-patholological features* (*Table I*)

Patients with carcinomas of homogenously poor differentiation were significantly ($P \approx 0.05$) younger ($55 \pm 19$ years) than those with well-differentiated tumours ($71 \pm 4$ years). All homogenously well-differentiated carcinomas were localized (Dukes' stage A and B), whereas most poorly differentiated ones were disseminated (Dukes' stage C and D). Histological grade showed no apparent relation to sex or tumour site. Six of the moderately differentiated tumours were disseminated (Table I).

### *Well-differentiated tumours*

All 6 homogenously well-differentiated tumours showed a heterogeneous staining pattern for HLA-DR antigens (Table I). In patient No. 3 the tumour was predominantly negative, whereas the remaining 5 expressed a strikingly patchy or variegated pattern (Figure 1). The staining for CEA was more homogenous (Table I). Four of the 5 tumours subjected to FCM showed a ND DNA profile.

### *Moderately differentiated tumours*

The expression of HLA-DR antigens tended to be more homogenous in the moderately-differentiated tumours than in the well-differentiated ones; two were uniformly negative, 14 showed one predominant staining pattern (Figure 2), and only 4 showed a strikingly variegated pattern (Table I). Intratumoural variation of the CEA staining was present in 9/20 moderately-differentiated carcinomas, but the variation was always small and did not exceed one fluorescence score (Table I). Fifteen of the moderately-differentiated tumours had an AN DNA profile.

**Table I** Clinico-pathological information about the patients, epithelial expression of HLA-DR antigens and CEA, and DNA ploidy within the different histological grades and Dukes' stages

| | *Patient no.* | *Age/Sex* | *HLA-DR Score** | *CEA Score** | *DNA ploidy* | *Dukes' stage* | *Tumour site* |
|---|---|---|---|---|---|---|---|
| Poorly diff. | 1 | 65 M | 0–3 | **1.5** | — | A | Sigmoid flexure |
| | 2 | 69 M | 0–3 | 1–1.5 | AN | A | Sigmoid flexure |
| | 3 | 76 F | **0**–1 | **1.5** | ND | A | Rectum |
| | 4 | 74 F | 0–3 | **1.5** | ND | A | Ascending colon |
| | 5 | 72 F | 0–3 | **2** | ND | B | Coecum |
| | 6 | 70 F | 0–1.5 | 1.5–2 | ND | B | Rectum |
| Moderately differentiated | 7 | 28 F | **0**–3 | **2** | AN | A | Transverse colon |
| | 8 | 55 M | **0**–2 | **2** | AN | A | Coecum |
| | 9 | 66 M | 0–1–2 | **1.5** | AN | B | Sigmoid flexure |
| | 10 | 52 M | 0–**1** | 1.5–**2** | AN | B | Descending colon |
| | 11 | 73 M | 1–**1.5** | **1.5** | AN | B | Rectum |
| | 12 | 78 F | 1–**1.5**–2 | 1.5–**2** | AN | B | Sigmoid flexure |
| | 13 | 69 M | **0**–0.5 | 1.5–2 | AN | B | Ascending colon |
| | | | 0–1–1.5 | 1.5–2 | AN | B | Rectum |
| | 14 | 28 F | 0.5–2 | 1–2 | AN | B | Coecum |
| | 15 | 52 M | 0–0.5 | **2** | ND | B | Ascending colon |
| | 16 | 54 M | 1–**2** | 1.5–2 | ND | B | Rectum |
| | 17 | 66 F | 1–**2** | 1–2 | ND | B | Rectum |
| | | | 1–2 | 1.5–2 | ND | B | Rectum |
| | 18 | 63 F | **0** | **2** | AN | B | Rectum |
| | 19 | 68 M | 0–3 | **2** | AN | C | Sigmoid flexure |
| | 20 | 67 F | **0**–3 | **3** | AN | C | Ascending colon |
| | 21 | 75 M | **0**–0.5 | **3** | AN | C | Rectum |
| | 22 | 69 F | **0** | **2** | AN | C | Rectum |
| | 23 | 70 M | **0**–1 | 1.5–**2** | AN | D | Transverse colon |
| | 24 | 47 F | 0.5–**1** | **2** | ND | D | Rectum |
| Well diff. | 25 | 29 M | **0** | **2** | AN | B | Rectum |
| | 26 | 39 F | **2** | **1** | AN | B | Ascending colon |
| | 27 | 45 F | **3** | **1** | AN | C | Coecum |
| | 28 | 54 F | **0.5** | **1.5** | AN | C | Sigmoid flexure |
| | 29 | 90 F | **2** | **0** | — | C | Sigmoid flexure |
| | 30 | 68 M | **0** | **2** | AN | D | Sigmoid flexure |
| | 31 | 47 F | **2** | **1** | — | D | Ascending colon |

*The fluorescence score of the predominating staining pattern is indicated by bold face.

### *Poorly differentiated tumours*

All of the 7 poorly-differentiated carcinomas showed a homogenous staining pattern, both for HLA-DR and CEA (Figures 3 and 4, Table I). Two cases were completely negative for HLA-DR antigens (Figure 3) and one for CEA (Table I). All tumours subjected to FCM DNA quantitation in this group showed an AN DNA profile.

### *Combined evaluation of HLA-DR and CEA expression in relation to tumour aggressiveness*

Most (87%) of the localized carcinomas (Dukes' stages A and B) showed heterogeneous expression of one or both epithelial markers (Table II).

**Table II** Localized and disseminated tumours categorized (percentage) according to staining pattern for HLA-DR and CEA

| *Tumour stage* | *Heterogeneous expression of both markers* | *Heterogeneous expression of one of the markers* | *Homogeneous expression of both markers* |
|---|---|---|---|
| Dukes' stages A and B ($n=22$) | 46% | 41% | 14% |
| Dukes' stages C and D (n = 11) | 9% | 36% | 55% |

**Figure 1** Well-differentiated near diploid (ND) large bowel carcinoma (patient no. 4). (a) Routine staining shows tubulo-villous structures with moderate atypia. (b) DNA profile is clearly ND. Diploid lymphocyte (LY) control indicated by arrow. (c) HLA-DR expression is patchy, varying from negative (bottom) to intensely positive (top). (d) CEA staining in same field is more homogeneous and confined to an apical rim. (a) ×200, (c) and (d) ×135.

**Figure 2** Moderately-differentiated distinctly aneuploid (AN) large bowel carcinoma (patient no. 8). (a) Routine staining shows glandular structures with severe atypia. (b) DNA profile shows distinct AN peak in addition to a near diploid one. Diploid lymphocyte (LY) control indicated by arrow. (c) HLA-DR expression is positive only in scattered clusters of epithelial cells. Note numerous HLA-DR$^+$ elements in stroma. (d) CEA staining in same field shows uniform apical rim with cytoplasmatic extensions. Glandular content is also positive. (a) ×200, (c) and (d) ×135.

**Figure 3** Poorly-differentiated distinctly aneuploid (AN) large bowel carcinoma (patient no. 30). (a) Routine staining shows the transition from adjacent colonic crypts (left) to anaplastic tumour elements (right). (b) DNA profile shows distinct AN peak in addition to a near diploid one. Diploid lymphocyte (LY) control indicated by arrow. (c) HLA-DR antigens are lacking within the tumour, with the exception of a few positive macrophages: the adjacent transitional crypt epithelium expresses HLA-DR determinants. Note numerous positive elements in the connective tissue. (d) CEA staining in same field is throughout the tumour, and adjacent crypts are moderately positive. (a) ×200, (c) and (d) ×135.

**Figure 4** Poorly-differentiated large bowel carcinoma (patient no. 31). (a) Routine staining. (b) HLA-DR expression is uniform and intense throughout the tumour. Note numerous positive elements in the stroma. (a) and (b) ×135.

Conversely, 55% of the disseminated tumours (Dukes' stages C and D) showed homogenous expression for both HLA-DR and CEA and 91% showed homogenous expression of at least one of the two markers (Table II).

*Non-neoplastic tissue elements*

The crypt epithelium in the transitional mucosa immediately adjacent to the tumours was always positive for both HLA-DR and CEA; this was so also in cases where the tumour epithelium was negative, regardless of grade of tumour differentiation. The fluorescence staining scores obtained in this zone (data not presented) showed no apparent relation to any of the variables investigated. Normal colonic epithelium was virtually unstained.

The endothelium of vessels in the tumour stroma was often stained for HLA-DR antigen. In addition, there was a large number of stained histiocytes located around the epithelial elements; a few such cells scattered within the tumours likewise seemed to represent HLA-DR$^+$ macrophages (Figures 2 and 3).

### Discussion

More than 90% of the large bowel carcinomas included in our study were positive for HLA-DR antigen—~70% showing a patchy staining pattern. Daar *et al.* (1982) described patchy expression of HLA-DR in 8/15 colorectal carcinomas, the remainders being negative. The positive patches were reported to amount to 10–15% of the examined tumour area and no correlation was found with the degree of tumour differentiation.

The discrepant results might be due to different antibody specificity and test sensitivity. We used a 3-step immunofluorescence technique applied to ethanol-fixed sections. The primary murine monoclonal antibody was directed against a non-polymorphic determinant and produced the same staining pattern in small intestinal epithelium as that obtained previously in our laboratory by the use of a polyclonal rabbit antiserum to human HLA-DR antigen (Scott *et al.*, 1980). Daar *et al.* (1982) used frozen sections and another monoclonal antibody in a peroxidase- anti-peroxidase technique. Thompson *et al.* (1982) found positive staining in 7/9 colon carcinomas by means of immunoperoxidase staining on frozen sections. They claimed that metastatic tumours were consistently HLA-DR$^+$ and showed a more widespread staining reaction than localized tumours. However, the small number of carcinomas examined might explain the failure to find negative metastatic tumours. The strikingly discrepant finding of Natali *et al.* (1981) indicating that undifferentiated tumours were consistently HLA-DR$^-$ might likewise be ascribed to inclusion of a few cases and perhaps differences in staining sensitivity.

In our study we found a remarkable relation between histological grade and the staining pattern for epithelial HLA-DR: homogenously well-differentiated tumours showed the greatest intra-tumour variability, moderately-differentiated tumours usually (80%) revealed one predominant staining pattern; whereas the poorly-differentiated ones were quite homogenously stained. A similar but less distinct trend was seen for CEA expression. When the staining patterns of the 2 epithelial markers were considered together, a homogenous expression of one or both antigens was clearly associated with increasing aggressiveness in terms of tumour dissemination (Table II).

These observations are in agreement with the concept of clonal evolution of tumour cell populations proposed by Nowell (1976). According to this hypothesis tumour progression is due to an acquired genetic lability permitting stepwise selection of variant sublines of neoplastic cells. The first malignant cell may build up early solid tumours by giving rise to several clones with different genotypic and phenotypic properties. Over time most variants will die but one clone may possess selective advantages and thereby emerge as a predominant subpopulation.

Most of the well-differentiated tumours had a ND DNA profile. One may postulate, therefore, that carcinomas of this category usually contain a variety of cell lines with approximately the same DNA content but with different biological properties including variable HLA-DR expression. The DNA data further indicated that in the development of tumours with a high malignant potential, distinctly AN clones of poor differentiation may arise and become increasingly dominant. such emergence of monoclonality could explain the remarkably homogeneous HLA-DR staining—either negative or positive—shown by the group of poorly-differentiated tumours. Nevertheless, disseminated tumours were sometimes ND (e.g., No. 24 in Table I) although preliminary clinical follow-ups have indicated that they give rise to a more protracted clinical course than the AN counterparts (Wolley *et al.*, 1982; Rognum *et al.*, 1983).

Our tentative conclusion is that well-differentiated tumours with a low malignant potential are heterogeneous with regard to HLA-DR expression and tend to be ND, wheras clonal selection taking place in highly malignant tumours may favour homogeneous expression of HLA-DR and CEA. Selection of particularly aggressive clones is usually associated with a distinctly AN DNA profile.

Although an AN DNA profile seems to be an unfavourable prognostic feature (Wolley *et al.*, 1982; Rognum *et al.*, 1983), the significance of the various patterns of HLA-DR and CEA expression remains unclear in terms of patient survival. We have previously shown that CEA tends to produce the strongest staining in moderately-differentiated large bowel carcinomas (Rognum *et al.*, 1982*a*).

The fact that highly malignant large bowel carcinomas were associated with relatively low age is in accordance with previous survival studies (Becio & Bussey, 1965), but the age-dependent endogeneous factors favoring tumour aggressiveness are unknown.

We found abundant staining for HLA-DR antigen in the crypt epithelium of the transitional mucosa adjacent to the tumours. This epithelium is probably subjected to stimulae from the carcinoma and may express HLA-DR as a reactive sign like activated T-cells (Hammerling, 1976) and macrophages (Steinman *et al.*, 1980). In graft-versus-host disease, colonic epithelium of the rat likewise expresses such antigens (Mason *et al.*, 1981). In accordance with Scott *et al.* (1980) and Thompson *et al.* (1982) we found that normal colonic epithelium was virtually negative or only faintly stained with the monoclonal antibody to HLA-DR antigens used in this study.

### References

ASHLEY, D.J.B. (1978). *Evan's Histological Appearance of Tumours*, 3rd edn. Edinburgh, Livingstone, p. 582.

BRANDTZAEG, P. (1974). Mucosal and glandular distribution of immunoglobulin components. Immunohistochemistry with a cold ethanol-fixation technique. *Immunology*, **26**, 1101.

BRANDTZAEG, P. & ROGNUM, T.O. (1983). Evaluation of tissue preparation methods and paired immunofluorescence staining for immunocytochemistry of lymphomas. *Histochem. J.*, **15**, 655.

DAAR, A.S., FUGGLE, S.V., TING, A. & FABRE, J.W. (1982). Anomalous expression of HLA-DR antigens on human colorectal cancer cells. *J. Immunol.*, **129**, 447.

GÖHDE, W. (1973). *Zellzyclusanalysen mit dem impulscytophotometer: Der Einfluss chemischer Noxen auf Prolieferationskinetik von Tumorzellen*. Münster, Thesis.

GÖHDE, W. & DITTRICH, W. (1971). Impulsfluorometri, ein neyarliges Durchflussverfahren zur ultraschnellen Mengenbestimmung von zellinhaltstoffen. *Acta Histochem.* (Suppl), **10**, 429.

HAMMERLING, G.J. (1976). Tissue distribution of 1a antigens and their expression on lymphocyte subpopulations. *Transplant. Rev.*, **30**, 64.

MASON, D.W., DALLMAN, M. & BARCLAY, A.N. (1981). Graft-vs-host disease induces expression of 1a antigens in rat epidermal cells and gut epithelium. *Nature*, **293**, 150.

NATALI, P.G., DEMARTINO, C., QUARANTA, V., BIGOTTI, A., BELLEGRINO, M.A. & FERRONE, S. (1981). Changes in Ia-like antigen expression on malignant human cells. *Immunogenetics*, **121**, 413.

NOWELL, P.C. (1976). The clonal evolution of tumour cell populations. Acquired genetic lability permits stepwise selection of variant sublines and underlies tumor progression. *Science*, **194**, 23.

RECIO, P. & BUSSEY, H.J.R. (1965). The pathology and prognosis of carcinoma of the rectum in the young. *Proc. R. Soc. Med.*, **58**, 789.

ROGNUM, T.O., BRANDTZAEG, P., ØRJASAETER, H., ELGJO, K. & HOGNESTAD, J. (1980). Immunohistochemical study of secretory component, secretory IgA and carcinoembryonic antigen in large bowel carcinomas. *Pathol. Res. Pract.*, **170**, 126.

ROGNUM, T.O., ELGJO, K., BRANDTZAEG, P., ØRJASAETER, H. & BERGAN, A. (1982*a*). Plasma carcinoembryonic antigen concentrations and immunohistochemical patterns of epithelial marker antigens in patients with large bowel carcinoma. *J. Clin. Pathol.*, **35**, 922.

ROGNUM, T.O., THORUD, E., ELGJO, K., BRANDTZAEG, P., ØRJASAETER, H. & NYGAARD, K. (1982*b*). Large-bowel carcinomas with different ploidy, related to secretory component, IgA and CEA in epithelium and in plasma. *Br. J. Cancer*, **45**, 921.

ROGNUM, T.O., THORUD, E., ØRJASAETER, H., DAHL, E. & HOGNESTAD, J. (1983). Clinical significance of different DNA ploidy patterns in large bowel carcinoma. *Abstract for the XIIth Meeting of European Study Group for Cell Proliferation*, Budapest, May 1983.

SCOTT, H., SOLHEIM, B.G., BRANDTZAEG, P. & THORSBY, E. (1980). HLA-DR-like antigens in the epithelium of the human small intestine. *Scand. J. Immunol.*, **12**, 77.

STAVE, R. & BRANDTZAEG, P. (1977). Fluorescence staining of gastric mucosa. A study with special reference to parietal cells. *Scand. J. Gastroenterol.*, **12**, 885.

STEINMAN, R.M., NOGUEIRA, N., WITMER, M.D., TYDINGS, J.D. & NEILMAN, I.S. (1980). Lymphokines enhances the expression and synthesis of Ia antigens on cultured mouse peritoneal macrophages. *J. Exp. Med.*, **152**, 1248.

THOMSON, J.J., HERLYN, M.F., ELDER, D.E., CLARC, W.H., STEPLEWSKY, Z. & KOROWSKI, H. (1982). Expression of DR antigens in freshly frozen human tumors. *Hybridoma*, **1**, 161.

TURNBULL, R.B., KYLE, K., WATSON, F.R. & SPRATT, J. (1967). Cancer of the colon: the influence of the no-touch isolation technique on survival rates. *Ann. Surg.*, **166**, 20.

VINDELOV, L.L., CHRISTENSEN, I.J., JENSEN, G. & NISSEN, N.T. (1983). Limits of detection of nuclear DNA abnormalities by flow cytometric DNA analysis. Results obtained by a set of methods for sample-storage, staining and internal standardization. *Cytometry*, **3**, 332.

WOLLEY, R.C., SCREIBER, K., KOSS, L.G., KARAS, M. & SHERMAN, A. (1982). DNA distribution in human colon carcinomas and its relationship to clinical behavior. *J. Natl Cancer Inst.*, **69**, 15.